# Oesophageal fluoroscopy in adults—when and why?

**DOI:** 10.1093/bjr/tqae062

**Published:** 2024-03-28

**Authors:** Martina Scharitzer, Peter Pokieser, Olle Ekberg

**Affiliations:** Department of Biomedical Imaging and Image-Guided Therapy, Medical University of Vienna, Waehringer Guertel 18-20, 1090 Vienna, Austria; Teaching Center, Medical University of Vienna, Waehringer Guertel 18-20, 1090 Vienna, Austria; Department of Translational Medicine, Diagnostic Radiology, Lund University, Skåne University Hospital, Inga Marie Nilssons gata 49, 205 02 Malmö, Sweden

**Keywords:** oesophagus, fluoroscopy, oesophageal motility disorder, oesophageal stenosis, diagnostic imaging

## Abstract

Oesophageal fluoroscopy is a radiological procedure that uses dynamic recording of the swallowing process to evaluate morphology and function simultaneously, a characteristic not found in other clinical tests. It enables a comprehensive evaluation of the entire upper gastrointestinal tract, from the oropharynx to oesophagogastric bolus transport. The number of fluoroscopies of the oesophagus and the oropharynx has increased in recent decades, while the overall use of gastrointestinal fluoroscopic examinations has declined. Radiologists performing fluoroscopies need a good understanding of the appropriate clinical questions and the methodological advantages and limitations to adjust the examination to the patient’s symptoms and clinical situation. This review provides an overview of the indications for oesophageal fluoroscopy and the various pathologies it can identify, ranging from motility disorders to structural abnormalities and assessment in the pre- and postoperative care. The strengths and weaknesses of this modality and its future role within different clinical scenarios in the adult population are discussed. We conclude that oesophageal fluoroscopy remains a valuable tool in diagnostic radiology for the evaluation of oesophageal disorders.

## Introduction

Oesophageal fluoroscopy is considered the primary imaging modality for a variety of oesophageal pathologies by supplementing, preceding, or replacing other investigation methods such as endoscopy or manometry. The use of oesophageal fluoroscopy has increased slightly, and videofluoroscopy or modified barium swallows have increased by more than 20% within the last 20 years.^1^ On a broader scale, diagnosing and treating swallowing disorders is a high priority, given global demographic trends with an increasingly ageing population. The prevalence of swallowing disorders in the general population is estimated to be around 20%, with higher rates in people over 50 years of age and an increased incidence in recent decades.^2^ In institutional healthcare, swallowing disorders affect up to 36% of older people admitted to hospitals and up to 50% in nursing homes.^3^ Therefore, instrumental swallow assessment by videofluoroscopy or fiberendoscopic evaluation of swallowing is required for diagnosis since clinical bedside tests show poor diagnostic performance and lack reliable and valid measurements.^4^

Due to the increasing availability of endoscopy and manometry, the spectrum of pathologies and referral patterns for oesophageal fluoroscopies have changed.^5^ This article aims to provide an overview of current indications of oesophageal fluoroscopy in adults and discuss the clinical relevance of radiological imaging.

## Basic overview of oesophageal fluoroscopy

As a non-invasive and cost-effective procedure,^6^^,^^7^ oesophageal fluoroscopy, whether a barium swallow or water-soluble swallow, is widely available. It has become a valuable initial test for patients with oesophageal symptoms that may guide further diagnostic procedures and medical, endoscopic, or surgical treatment decisions. Oesophageal fluoroscopy is usually performed as a multiphasic examination, including upright, prone, and supine positioning (Table 1). The radiological study should include the pharyngeal phase in the first swallowing sequence to detect pathologies such as aspiration. Regarding the choice of contrast medium, low-density barium enables good flow properties for easy oesophageal transport and sufficient opacity. In case of aspiration, an abbreviated protocol with an iodinated, non-ionic contrast medium may be performed. Water-soluble contrast medium is also recommended in cases with suspicion of an oesophageal leak. Recording and documenting the fluoroscopic sequences enables the assessment of the swallowing scenes in slow-motion and the subsequent frame-by-frame analysis. Recent innovations in fluoroscopic imaging have significantly reduced dose levels due to the increased sensitivity of flat panel detectors,^8^ alleviating radiation dose concerns.

**Table 1.** Different investigation components for evaluation of the oesophagus.

View	Anatomic region	Pathology investigated
Upright single-contrast (RPO/LPO)	Oropharynx Pharyngooesophageal segment Oesophagus, OGJ	Initial evaluation of oropharyngeal and oesophageal structure and function Bolus transit time
Supine and prone single contrast	Oesophagus, OGJ, stomach	Oesophageal function (primary peristaltic wave) Bolus transit time Strictures + rings, diverticula, other wall abnormalities
Double contrast	Oesophagus, OGJ, stomach	Hiatal hernia Strictures + rings, diverticula, other wall abnormalities
Prone right anterior oblique single and sequential swallows	OGJ Pharynx + oesophagus optionally	Hiatal hernia, cardia configuration Transient relaxation of OGJ, width of diaphragmatic entrance Strictures + rings
Additional views depending on previous findings	OGJ, stomach	Morphological and functional abnormalities Angle of His
Upright tablet test or solid food test	Oesophagus, OGJ, stomach	Obstruction, retention Trigger of symptoms, oesophageal hypersensitivity

Abbreviations: OGJ = oesophagogastric junction; RPO = right posterior oblique; LPO = left posterior oblique.

The American College of Radiology generally considers oesophageal fluoroscopy appropriate for oropharyngeal, retrosternal and postoperative dysphagia,^9^ and the ECR European training curriculum includes the skills of performing contrast studies of the entire swallowing mechanism independently.^10^

## General indications

The fluoroscopic examination of the oesophagus is used to assess the anatomy and physiology of deglutition and to determine the causes and severity of swallowing disorders by visualizing bolus flow and the involved structures along the upper gastrointestinal tract. General indications for oesophageal fluoroscopy include patients with dysphagia, globus sensation, non-cardiac chest pain, and symptomatic or suspected gastroesophageal reflux disease. As an operator depending procedure, the examination must be tailored to the patient’s needs and clinical symptoms, which is facilitated by careful history-taking before the investigation.^11^

Patients with dysphagia may undergo various diagnostic tests, such as a nose and throat examination for naso- and oropharyngeal pathologies, endoscopy for mucosal abnormalities, or manometry to assess motility disorders. In this group of patients, oesophageal fluoroscopy is a global primary test assessing morphological and functional disorders simultaneously.^12^ A significant number of patients with globus sensation, the feeling that something is stuck in the throat, which may improve during meals, have abnormalities of motor function and/or gastroesophageal reflux disease.^13^ Oesophageal fluoroscopy has been shown to reveal significantly more abnormalities compared to oropharyngeal videofluoroscopy alone and has high specificity for motility disorders compared to manometry.^14^ In patients with non-cardiac chest pain, gastrooesophageal reflux disease (GORD) is the most common cause, followed by functional chest pain with oesophageal hypersensitivity, oesophageal dysmotility, eosinophilic oesophagitis, and psychological comorbidity.^15^ In this patient group, fluoroscopy can help in the diagnosis or exclusion of oesophageal causes.

Care should be taken to ensure that any symptoms suggestive of a malignancy, such as rapidly developing dysphagia on solid foods and weight loss, require immediate endoscopy to avoid unnecessary time delays. In contrast, patients with oesophageal motility disorders and benign strictures often present with slowly progressing symptoms on liquids and solid foods or intermittent solid-food dysphagia.

There are only a few contraindications to performing oesophageal fluoroscopy. The patient should be in a stable state of health and conscious enough to be positioned in the fluoroscopy suite and to cooperate. The patient should be able to swallow at least small amounts of contrast medium.

**Figure 1.**
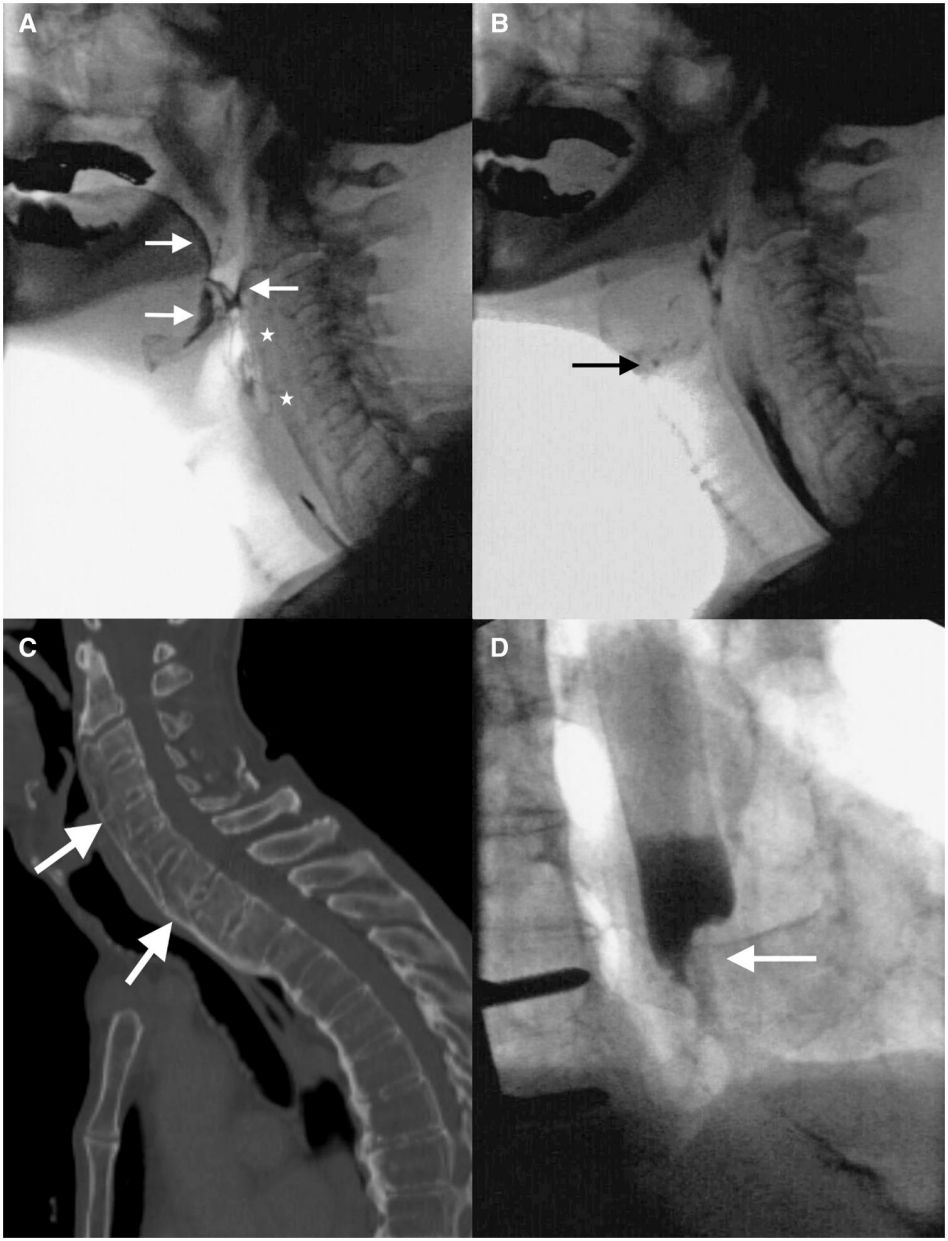
Combined pharyngeal and oesophageal pathology. (A) 78-year-old patient with dysphagia and globus sensation in the cervical region for 6 weeks. Fluoroscopy revealed diffuse idiopathic skeletal hyperostosis (asterisk) with postdeglutitive pharyngeal residues (arrows) due to pharyngeal weakness and (B) minimal deep penetration (arrow), which is a possible explanation for his symptoms. (C) CT shows the extent of ossification of the anterior longitudinal ligament (arrows). (D) Oesophageal assessment revealed irregular, asymmetric stenosis in the distal oesophagus (arrow) with transient delayed bolus passage. Subsequent endoscopy confirmed oesophageal carcinoma.

Accurate clinical differentiation between oropharyngeal and oesophageal dysphagia can be difficult. The location of where patients report their swallowing problems is often inaccurate, with one-third locating the source of their symptoms more proximally and to the cervical region, despite having an oesophageal aetiology as the underlying cause.^16^ Regarding the level of underlying pathology, obstructive lesions of the lower oesophagus are least likely to be correctly localized.^17^ The high percentage of patients with oesophageal dysphagia manifesting as pharyngeal symptoms underlines the value of a global radiological assessment from the pharyngeal phase to the clearance of oral contrast into the stomach (Figure 1). In addition, observation of the swallowing process close to the patient enables correlation between a fluoroscopic finding and the patient-reported onset of symptoms.

A second important factor is the frequent combination of oropharyngeal and oesophageal pathologies due to the close interrelationship between the oral, pharyngeal, and oesophageal swallowing continuum. A systematic review by Reedy et al. found prolonged oesophageal bolus transport in 49% of patients undergoing a modified barium swallow.^18^ O’Rourke et al. have used high-resolution manometry to demonstrate oesophageal alterations during voluntary pharyngeal manoeuvres, finding strong associations between the oropharynx and oesophagus.^19^ These interrelations underline the importance of understanding that swallowing is a process beginning with oral bolus intake and ending with entry into the stomach or even beyond.

**Figure 2.**
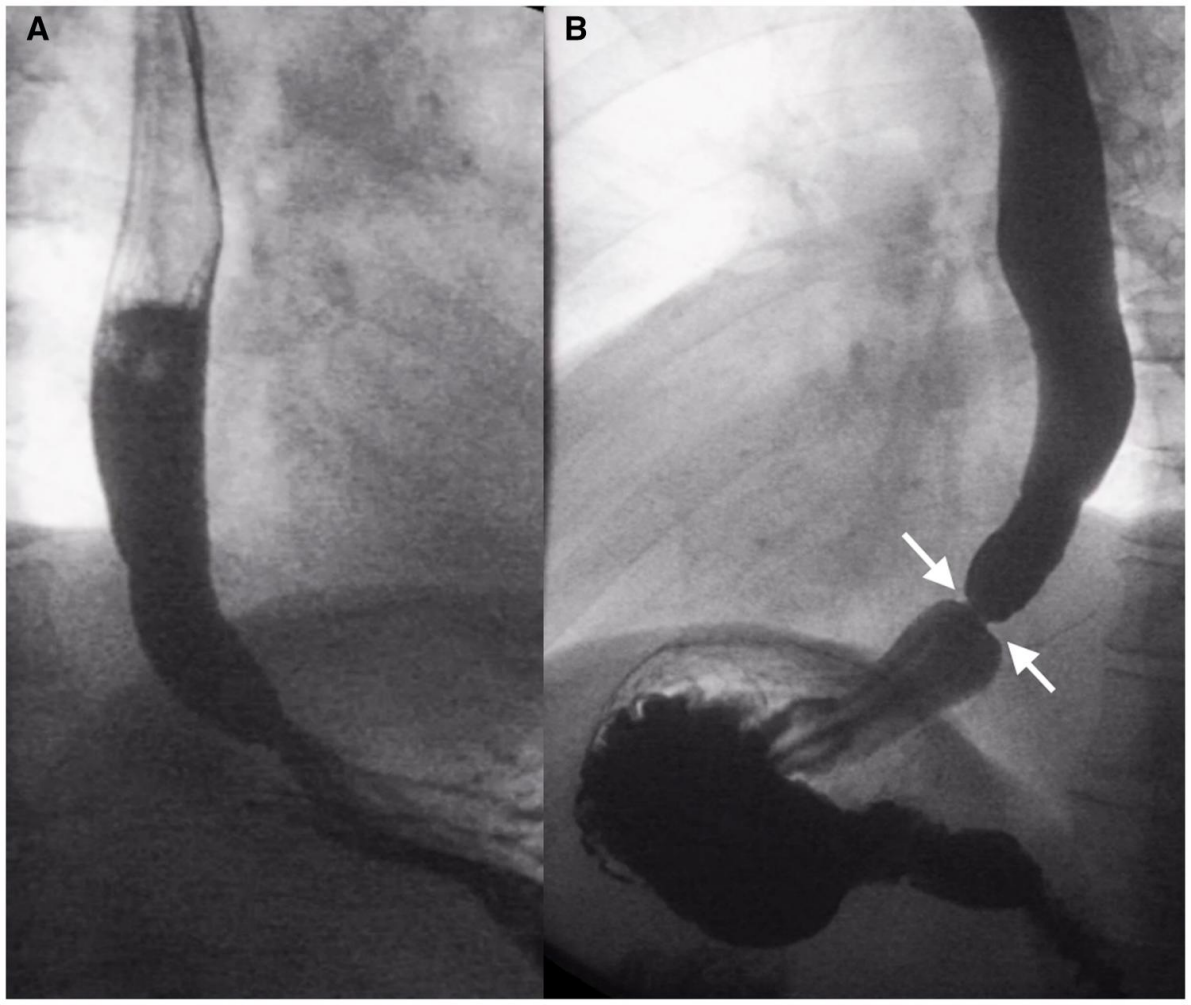
Importance of oesophageal distension in the assessment of rings. (A) Upright left posterior oblique fluoroscopy in a 57-year-old patient with recurrent bolus impactions demonstrated timely bolus passage into the stomach. (B) Distension of the oesophageal lumen in the prone right anterior oblique position revealed a smooth, symmetric, annular mucosal ring (arrows) in the distal oesophagus with significant lumen narrowing, which was not visible in standing position with inadequate distension.

To overcome these diagnostic difficulties and with an increasing number of non-radiological staff performing videofluoroscopies/modified barium swallows of the oropharynx, a sweep protocol has been established. This approach includes an abbreviated routine visualization of the oesophagus by speech-language therapists including two to three single swallows in standing position. Using an oesophageal sweep, Watts et al. found a combination of oropharyngeal and oesophageal dysfunction or primarily oesophageal pathologies in 26% of patients.^20^ However, findings were not verified, and the number of missed pathologies was not assessed. In contrast, Allen et al. compared oesophageal screen findings with full oesophagrams and found limited screening sensitivity compared to a comprehensive oesophagram evaluation^21^ (Figure 2). Therefore, as stated by Ekberg, the cursory evaluation of oesophageal bolus transit time should be interpreted with knowledge of its limitations and not be relied upon to assess oesophageal morphology and function.^22^

## Specific clinical questions

The following sections discuss specific clinical scenarios and situations to highlight the current evidence-based strengths and limitations of oesophageal fluoroscopy.

### Oesophageal motility disorders

Radiologic evaluation of oesophageal function has become a widely accepted diagnostic method for motility disorders based mainly on subjective interpretation of observational findings. It can be assessed in real-time, although videotaping enables repeated analysis of the bolus passage. Compared with oesophageal manometry, it has variable sensitivity, being highest in achalasia.^23^

A thorough fluoroscopic investigation includes the assessment of the oesophageal body, also covering the pharyngooesophageal segment and the lower oesophageal sphincter. Peristalsis is assessed in the upright and prone oblique position, the latter of which is mandatory to evaluate oesophageal function without support from gravity. Regular swallowing induces a primary peristaltic contraction wave with a usual fluoroscopic appearance of an inverted “V”-shape of the bolus tail. The proximal escape of small amounts of barium and the “deglutitive inhibition”, reflecting inhibition of oesophageal peristalsis during swallowing in rapid succession,^24^ are considered not pathological. A residual bolus or gastrooesophageal reflux activates a secondary peristaltic wave triggered by oesophageal distension, which is also considered a normal pattern.

Significant technical advances such as high-resolution manometry and an endoscopic impedance planimetry system measuring oesophageal pressure and dimensions, called the functional luminal imaging probe, have improved the characterization of oesophageal motility disorders. They are classified according to the Chicago classification v4.0, based on manometry findings (Table 2). Disorders of peristalsis include absent contractility, distal oesophageal spams, hypercontractile oesophagus, and ineffective oesophageal motility. The latter encompasses weak contraction, failed peristalsis, or a fragmented swallow with a transition zone defect of peristalsis.^24^ Ineffective motility can be seen fluoroscopically as a loss of peristaltic continuity and segmental incomplete contractions without a propagating effect, which may arise at several sites and can occur repeatedly. In patients with diffuse oesophageal spasm, severe nonperistaltic contractions, defined by intermittently abnormal peristalsis and obliteration of the lumen, can be seen, despite the majority of patients only having moderate nonperistaltic contractions.^25^ A typical beak-like aspect of the oesophagogastric junction (OGJ) with absent oesophageal motility suggests achalasia. Patients with scleroderma may exhibit absent contractility in the lower oesophagus, which can be observed as a complete lack of any visible oesophageal motility.

**Table 2.** Chicago Classification version 4.0 for oesophageal motility disorders and corresponding radiological findings at oesophageal fluoroscopy.

Disorder	Manometric definition		Radiological findings			Comments
Disorders of the OGJ outflow = abnormal relaxation pressure at OGJ						
Type I achalasia	100% failed peristalsis	No pan-oesophageal pressurization	Oesophageal dilation Tortuous/sigmoid appearance Aperistalsis	Bird’s beak of OGJ* Reduced bolus flow across the OGJ* Stasis (prone position)* Support level (upright position)*		TBE enables quantification
Type II achalasia		+ ≥20% swallows with pan-oesophageal pressurization	Lesser dilation and lesser delayed emptying compared to type I			Lower sensitivity for diagnosis of type II + III compared to type I achalasia Tablet test increases sensitivity at cost of reduced specificity Normal fluoroscopy does not exclude achalasia Fluoroscopy may secure diagnosis in inconclusive manometry Valuable for assessing therapeutic response
Type III achalasia		+ ≥20% swallows with premature/spastic contraction	Corkscrew appearance = repetitive, lumen-obliterating, nonperistaltic contractions			
OGJ outflow obstruction	≥20% elevated intrabolus pressure, not meeting criteria for achalasia		Fluoroscopy can rule out structural OGJ obstruction			

Disorders of peristalsis = normal relaxation pressure at OGJ						
Absent contractility	100% failed peristalsis		Absent oesophageal stripping wave			
Distal oesophageal spasm	≥20% swallows with premature/spastic contraction		Corkscrew appearance (=rosary bead, spasm) Many non-propulsive contractions in distal oesophagus			Radiologic findings often nonspecific
Hypercontractile oesophagus	≥20% hypercontractile swallows		Usually normal fluoroscopy, preserved peristalsis Minority may show nonspecific oesophageal motility disorder			
Ineffective oesophageal motility	≥70% ineffective swallows or ≥50% failed peristalsis		Loss of peristaltic continuity Segmental incomplete contractions without a propagating effect			Non-propulsive contractions can also be seen in healthy, especially older patients

Abbreviations: OGJ = oesophagogastric junction; TBE = timed barium oesophagram.

Classification of oesophageal motility disorders is based on manometric findings. Radiologically, functional disorders are characterized by a disruption of the primary peristalsis and prolonged oesophageal bolus clearance. The presented radiological findings represent typical corresponding appearances on oesophageal fluoroscopy, although a wide overlap exists between these disorders.

* These radiological signs can be found in all disorders of OGJ outflow.

However, radiographic studies may not detect intermittent contraction abnormalities reliably, leading to a reported sensitivity of 73%.^26^ Still, quantitative normative fluoroscopic measures are lacking. Regular oesophageal bolus transport in healthy individuals of different ages needs to be sufficiently defined. Usually, a peristaltic wave passes a bolus at a speed of about 2 cm/s and should not take longer than 15 s for oesophageal bolus passage in lying position.^27^ Uncertainty in the diagnosis of a delayed passage might arise due to the influence of bolus consistency and volume and a normal range of prolonged transport and intra-oesophageal stasis in the ageing population.^28^ These cases highlight the need for a comprehensive evaluation of other fluoroscopic pathologies. Adding a tablet of standardized size or a solid bolus test to the examination to assess emptying time increases accuracy.^29^

In the timed barium oesophagram (TBE), images are taken 1 min and 5 min after ingesting 200–250 mL of barium suspension.^30^ Oesophageal retention is quantified by measuring the height of the retained contrast medium above the OGJ with thresholds of 5 cm at 1 min and 2 cm after 5 min.^31^ This test has shown promising results in detecting oesophagogastric outlet obstruction and predicting response to treatment in achalasia.^32^ However, the wide range of barium column height and width on single radiographs requires caution, especially in achalasia type II and III with pan-oesophageal pressurisation and spastic contractions.^33^ If the height of the contrast column at baseline was less than 10 cm, repeat examination to assess response to treatment is not recommended because of the relatively large daily variability.^34^ A distinction between anatomic obstruction and functional oesophagogastric outlet obstruction is impossible,^35^ underlining the need for a thorough oesophageal radiological examination.

In patients with non-obstructive dysphagia and suspicion of delayed bolus passage, oesophageal fluoroscopy may reveal successful clearance, supporting the diagnosis of oesophageal hypersensitivity and disordered perception, and may help rule out other pathologies.^36^

### Gastrooesophageal reflux disease

As a complex barrier between the thoracic and abdominal intestinal compartments, the OGJ is challenging to investigate due to the mobile relationship between the distal oesophagus, the hiatus, and gastric cardia. According to ACG guidelines, a barium swallow study is not recommended solely as a diagnostic modality for gastrooesophageal reflux assessment^37^ since the presence of gastrooesophageal reflux during a fluoroscopic study has shown poor sensitivity and specificity compared to pH-testing.^38^ If spontaneous reflux after turning the patient from the supine to the prone position cannot be observed, additional provocative tests such as a straight leg-raising, a Valsalva manoeuvre to increase intraabdominal pressure, or the water-siphon test (continuously drinking water in the supine right posterior oblique [RPO] position) can be performed to increase the likelihood of detecting gastrooesophageal reflux of contrast medium. The use of these tests is viewed controversial, as specificity decreases with increasing sensitivity,^39^ which is also addressed in the consensus statement of the “Society of Abdominal Radiology disease-focus panel on GORD.”^39^

**Figure 3.**
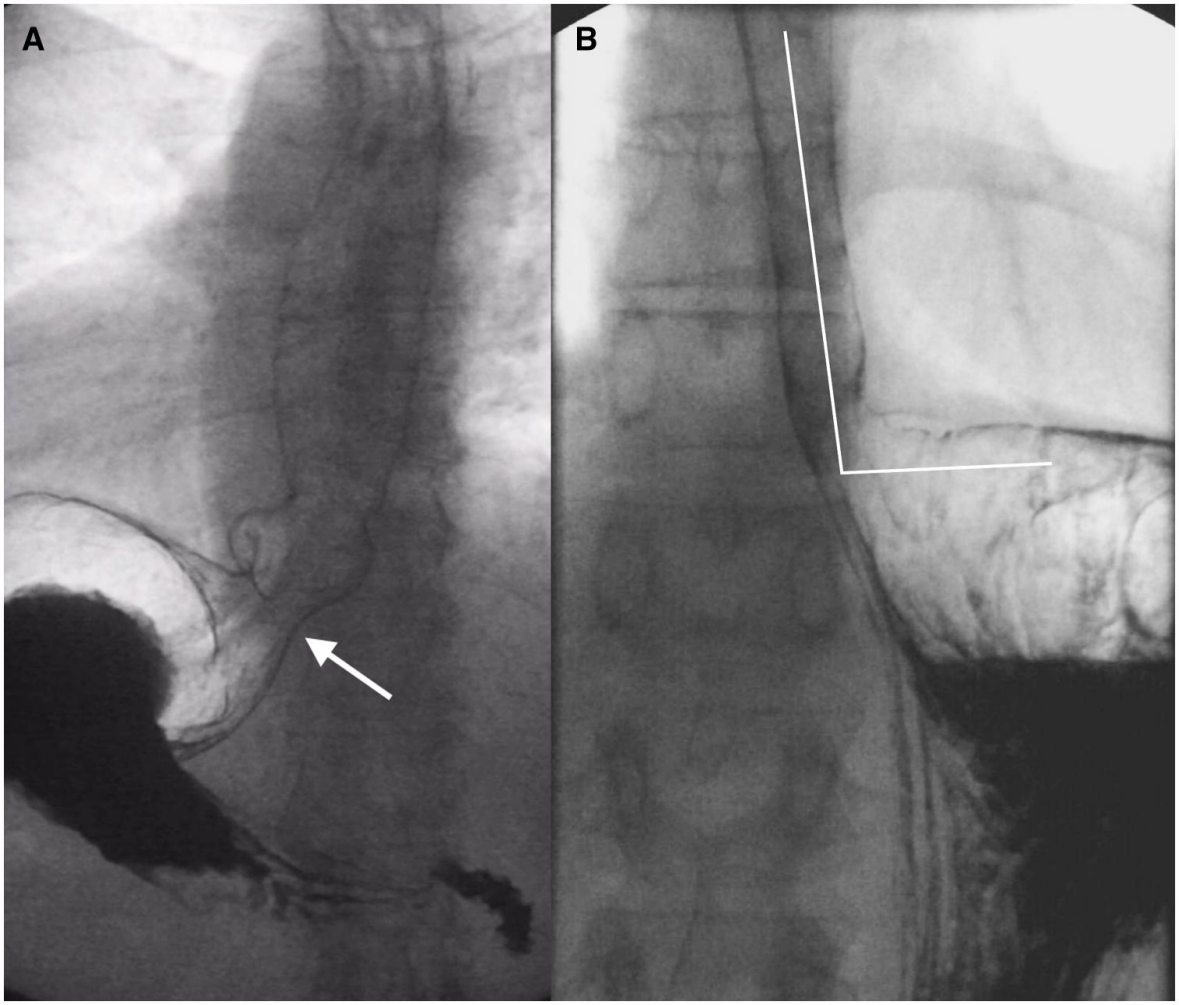
Two different patients with symptoms of gastrooesophageal reflux. (A) Fluoroscopy revealed laxity of ligamentous attachments leading to insufficiency of the cardia with a funnel-shaped configuration (arrow) and a large diaphragmatic hiatus. (B) In a different patient, the angle of His increased after repositioning from the recumbent to the upright position. It should be acute and not obtuse.

There are other radiological signs supporting the diagnosis of GORD. Oesophageal dysmotility is significantly more often associated with gastrooesophageal reflux than with patients with presbyoesophagus presenting with multiple nonperistaltic contractions.^40^ Weakening of the phrenooesophageal ligament may result in an incompetence of the OGJ. Additional signs suggestive of GORD^41^^,^^42^ are a change of the cardia configuration,^43^ widening of the OGJ,^44^ formation of a hiatal hernia and widening of the Angle of His, the insertion angle formed by the longitudinal axis of the lower oesophagus and the tangent to the right side of the gastric fundus^45^ (Figure 3). Since signs of reflux oesophagitis are typically found proximally to the gastrooesophageal junction, a reticular mucosal pattern found at a considerable distance from the gastrooesophageal junction or the presence of a mid-oesophageal stricture are suggestive of Barrett dysplasia in patients with reflux disease and a hiatal hernia.^46^ Nevertheless, endoscopy is the gold standard for the assessment of reflux-induced oesophagitis.

### Structural oesophageal pathologies

A radiological swallowing examination often primarily assesses a benign and malignant structural narrowing of the oesophagus.^47^ Within the last decade, the spectrum of benign oesophageal stenoses has changed with less peptic strictures in the era of widespread antireflux therapy and more stenosis-like rings, webs, or a small-calibre oesophagus.^48^^,^^49^ Mucosal rings have been reported to occur in 15% to 26% of dysphagic patients.^50^ Fluoroscopy may be more sensitive than gastroscopy in finding moderate stenosis with a luminal diameter of more than 10 mm.^51^ This can be explained by the difficulty in achieving optimal distension during endoscopy and static single-spot radiography.

On the other hand, distal oesophageal rings may be missed due to overdistention above a hiatal hernia, producing an “overlap phenomenon”.^52^ In these cases, patients should swallow smaller volumes of contrast medium. In patients with a history of solid-food dysphagia, using a standardized tablet of 13–14 mm has been proven to estimate residual lumen diameter to guide further treatment accurately.^53^ The use of other solid food tests with barium-based bread, marshmallows, radiopaque tablets, or even magnetic disc tablets has been published to facilitate the diagnosis and estimation of true size of the residual oesophageal lumen diameter if the solid bolus stops or is delayed.^54–56^

**Figure 4.**
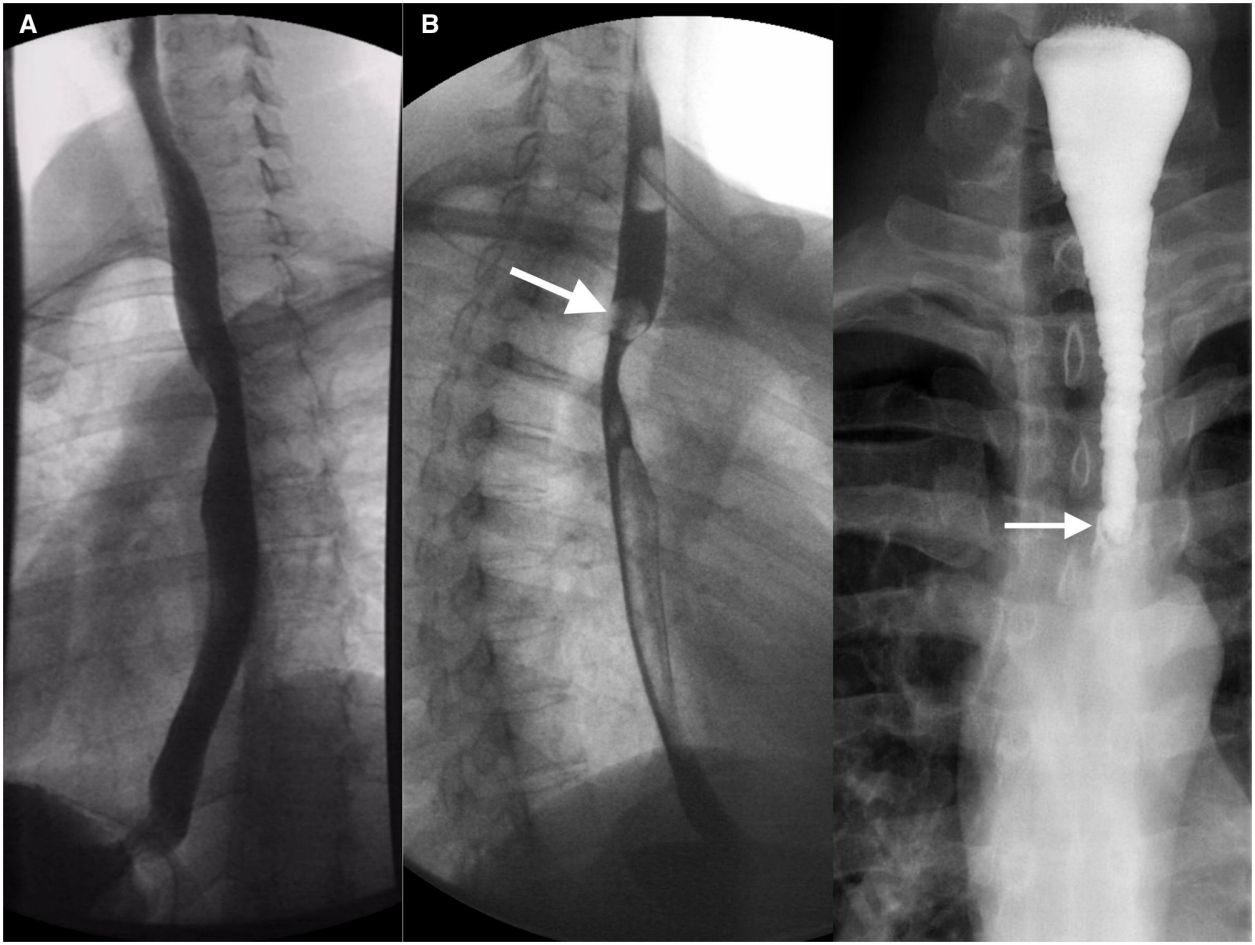
Eosinophilic oesophagitis in two patients. (A) Right anterior oblique fluoroscopic image revealed a long-standing reduction in the oesophageal calibre, the small-calibre oesophagus. (B) Fluoroscopic image after ingestion of a 14-mm tablet shows retention of the tablet (arrow) in the mid-oesophagus. (right image) Another patient presenting with solid-food dysphagia and food impaction (arrow) with several annular indentations in the proximal oesophagus, the so-called ringed oesophagus.

Eosinophilic oesophagitis, a chronic immune-mediated inflammatory disease, is a rapidly increasing cause of solid-food dysphagia in children and young adults, with a reported prevalence of around 34.4 cases/100,000 persons.^57^ Although the final diagnosis is made on endoscopic biopsy, oesophageal fluoroscopy may reveal characteristic findings highly suggestive of this entity: these include ring-like indentations, also called ringed oesophagus^58^ or trachealization^59^ and a segment of oesophageal narrowing longer than 8 cm and narrower than 20 mm, a small-calibre oesophagus^60^ (Figure 4). Gentile et al. stated that a loss of oesophageal distensibility is not easily detectable by endoscopy, with a reported sensitivity of 25% to identify residual oesophageal lumen ≤15 mm compared to oesophagography.^61^ Fluoroscopy is therefore a valuable tool for monitoring disease activity by showing the formation of clinically relevant strictures too subtle to be assessed by endoscopy.^57^

**Figure 5.**
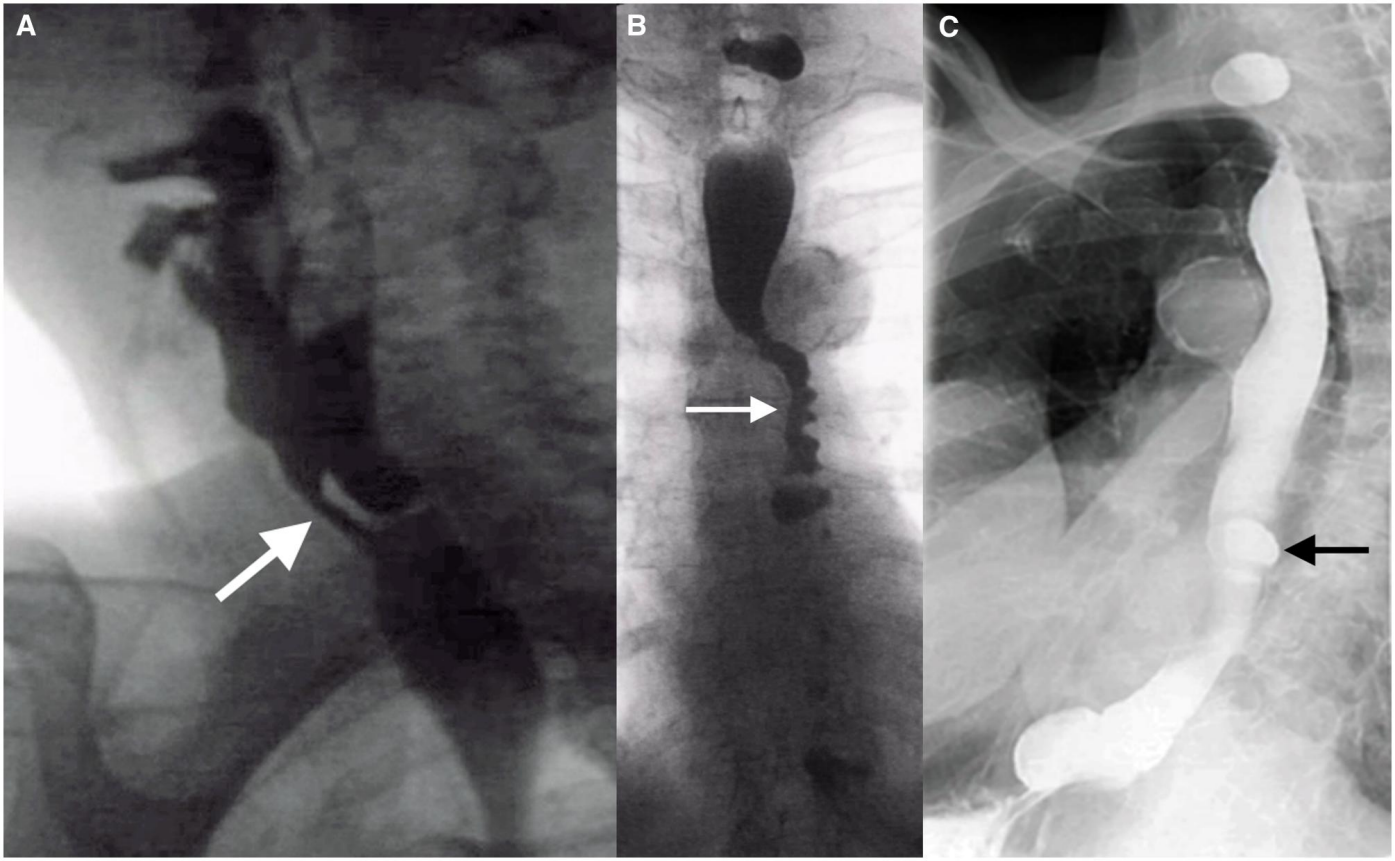
Combination of structural and functional pathologies. (A) Fluoroscopy in an 82-year-old patient with dysphagia for liquids and solids and regurgitation revealed a Zenker’s diverticulum with significant compression of the oesophageal lumen (arrow). (B) Evaluation of the oesophagus shows diffuse spastic contractions (arrow). (C) In recumbent, right anterior oblique position, a midoesophageal diverticulum is seen (arrow).

Oesophageal diverticula may occur in the pharyngooesophageal region (Zenker, Kilian-Jamieson), the mid-oesophagus, or distally (epiphrenic diverticulum) and are often associated with motility disorders. Preoperative oesophageal fluoroscopy showing the size of the diverticulum, its muscular septum, and its stenotic effect proved extremely important for surgical planning.^62^ When correlating dysphagia severity with fluoroscopic findings of a Zenker diverticulum, the maximum opening of the pharyngooesophageal segment was predictive of the severity of swallowing function (Figure 5). In contrast, diverticulum size and the bolus entry angle were not.^63^ The correlation of symptoms occurring during the investigation to associated fluoroscopic findings may facilitate the decision of whether structural failure or motility disorders are responsible for the patient’s symptoms.

### Assessment in comorbidities with high suspicion of oesophageal involvement

In connective tissue or rheumatologic disorders, the gastrointestinal tract is often affected. In patients with systemic sclerosis (SSc), up to 90% show oesophageal manifestation, with increased mortality rate in severe oesophageal involvement.^64^ Oesophageal fluoroscopic abnormalities are associated with pulmonary disease in children with juvenile SSc.^65^ Radiologic investigation as an initial test shows a dilated, air-filled distal oesophagus caused by absent peristalsis in the smooth muscles.^66^ Other systemic diseases with possible oesophageal involvement are mixed connective tissue disease, myositis, Sjogren’s syndrome, or systemic lupus erythematosus.

Diabetes mellitus may manifest as heartburn and reflux symptoms.^67^ In thyroid disease, patients may be referred to fluoroscopy having dysphagia or obstructive symptoms caused by direct compression of an enlarged gland. Rare systemic diseases with oesophageal involvement and swallowing disorders include genetic syndromes like Down syndrome, infiltrative disorders like amyloidosis, inflammatory conditions like Behcet’s disease, or neuromuscular diseases like multiple sclerosis.^68^ While these diseases are heterogeneous, knowing potential oesophageal manifestation is necessary when performing oesophageal fluoroscopy.

### Preoperative assessment

Preoperative radiological assessment contributes to selecting the most appropriate surgical technique and may reveal concomitant abnormalities, such as oesophageal motility disorders in GORD, which may require a different surgical approach. The Oesophageal Diagnostic Advisory Panel has stated that a radiological barium investigation is necessary before antireflux surgery.^59^ Assessing the oesophageal length may provide useful preoperative information for lengthening procedures. Diagnosing the presence and size of a hiatal hernia enables the planning of surgical reduction. Fluoroscopy and recording of video loops are preferable to allow visualization of temporal changes of dynamic oesophageal structures related to bolus flow, such as intermittent changes in the size of a hiatal hernia.^69^

### Postoperative assessment

Radiological evaluation of the oesophagus is essential in the follow-up of patients undergoing antireflux surgery or oesophageal resection to assess surgical effectiveness and detect postoperative complications. Water-soluble low-osmolar contrast agents are generally the contrast medium of choice in patients with possible gastrointestinal perforation, as they are absorbed quickly and without harmful effects from the extraluminal spaces. In the early postoperative period, oesophageal fluoroscopy is frequently used in clinical routine to rule out anastomotic leak, obstruction resulting from oedema or stenosis, or other rare complications like slipping, paraoesophageal hernia, or stasis due to vagotomy. On the one hand, controversies exist regarding its use as a screening method to confirm correct anastomotic patency, showing high specificity but a reported sensitivity of less than 50% for assessing occult anastomotic leak.^70^ On the other hand, estimated cost savings for immediate identification of an anastomotic leak are significantly higher than for patients with symptomatic leaks.^71^ The ACR considers an oesophagram the method of choice in early postoperative dysphagia.^9^ A systematic review has shown an overall anastomotic leak rate of 11% following oesophagectomies,^72^ whereas perforations discovered postoperatively in laparoscopic foregut surgery were found in 0.7%.^73^ Therefore, several clinicians propose to abstain from routine swallowing examinations but recommend an immediate implementation in patients with clinical suspicion of leakage.^74^ Due to the substantially higher specificity of fluoroscopy for detecting leaks, Lantos et al. showed the highest confidence in excluding oesophageal perforations when combining oesophagography with CT.^75^

**Figure 6.**
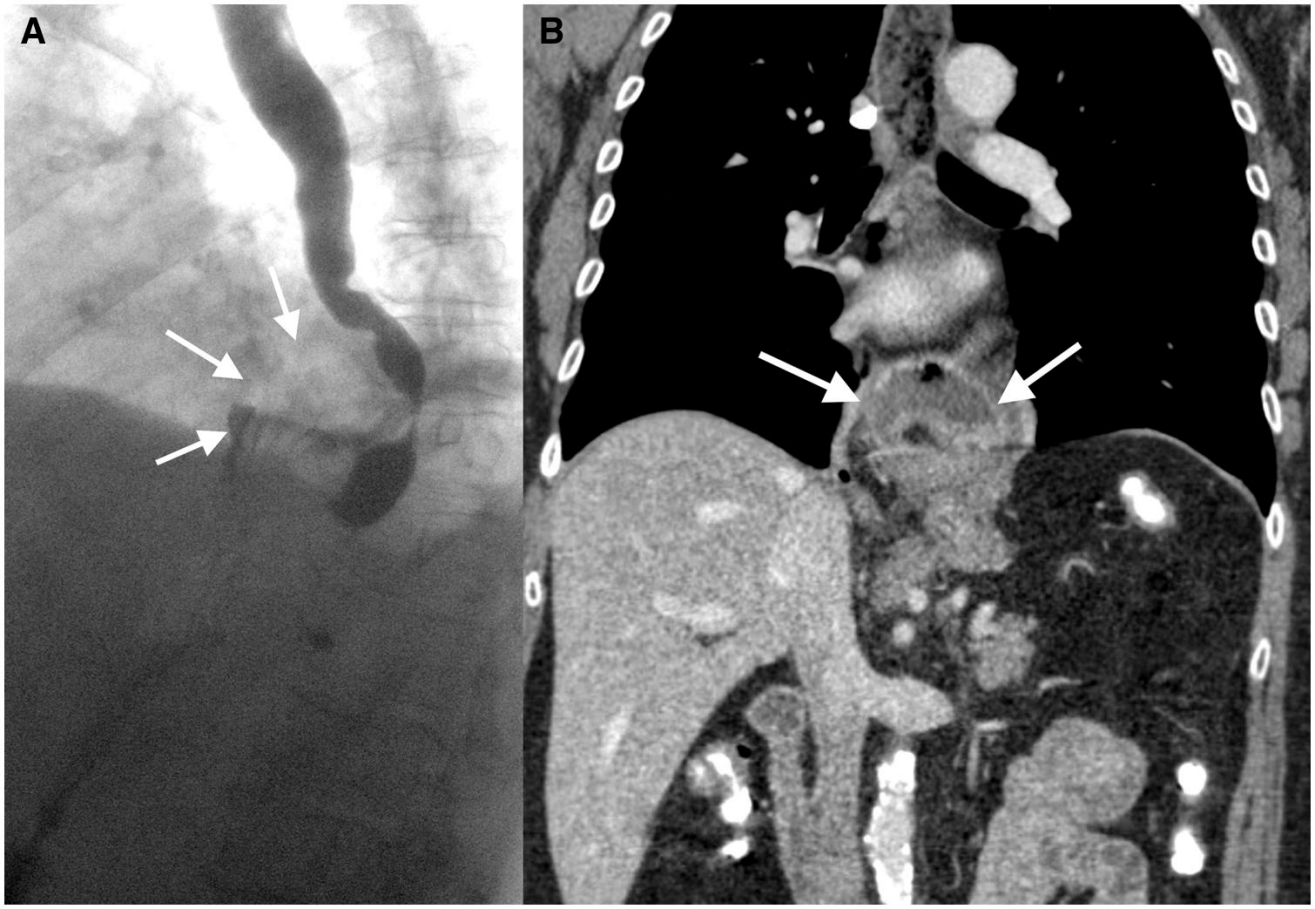
Supradiaphragmatic migration of a slipped stomach. (A) One day after Nissen fundoplication, fluoroscopy revealed paraoesophageal slipping of gastric parts into the supradiaphragmatic region (arrow). (B) CT confirmed paraoesophageal slipping of the fundoplication wrap (arrows).

Assessment of long-term complications includes diagnosis of recurrence of hernia formation, stenosis, or fistula formation. The latter can be examined particularly well under fluoroscopy, as the ability to reposition the patient under real-time fluoroscopy, as opposed to CT or MRI, aids in correct diagnosis. Signs of failed fundoplication include obstruction of the oesophageal lumen, recurrence of gastrooesophageal reflux, or impaired integrity or dislocation of the gastric wrap.^76^ Intrathoracic migration of a wrap or acute paraoesophageal hiatal hernia may remain silent^77^ but require immediate diagnosis and re-intervention^78^ (Figure 6). In patients with persistent stenosis at the OGJ after fundoplication, the percentage of time attributable to the OGJ transit, proximal escape of bolus, and peristaltic dysfunction correlated significantly with clinical dysphagia scores, whereas manometric parameters did not.^79^

Follow-up of patients after surgical repair of a Zenker diverticulum could not show prognostic factors regarding the recurrence of the disease.^62^ Similarly, early oesophagram after per-oral endoscopic myotomy for achalasia did not predict long-term outcomes.^80^

While reducing the risks of obesity-related comorbidity, bariatric surgery holds risks for the development of oesophageal dysfunction with increased gastrooesophageal residual pressures, incomplete relaxation, and increased peristaltic wave duration.^81^ The term post-obesity surgery oesophageal dysfunction (POSED), describing an achalasia-like pattern with aperistalsis and increased intragastric pressure, has become established and was found in 5% of postsurgical patients.^82^ Besides this motility disorder, fluoroscopy may show pouch dilatation with gastrojejunal anastomotic stenosis or obstruction at or related to the jejunojejunal anastomosis in symptomatic patients.^83^

### Acute setting

Foreign body impactions in the oesophagus are common emergencies requiring urgent evaluation and treatment, depending on institutional algorithms and the availability of specialists. A fluoroscopic investigation with low-osmolar water-soluble contrast medium can confirm the diagnosis and determine the level of obstruction to guide further management. However, national guidelines on the use of a radiographic evaluation differ.^84^ The ESGE Clinical Guideline proposes fluoroscopy in cases of an oesophageal foreign object not detectable on plain radiography, except if an oesophageal obstruction is suspected clinically.^85^ Radiological examinations should not cause delays in performing necessary endoscopic procedures. Fluoroscopy-guided oesophageal treatment of distally ingested food using intravenous glucagon, effervescent agent, and water has been described as a safe, effective, and cost-efficient alternative to endoscopy.^86^

Patients with vomiting, chest pain, and subcutaneous emphysema may suffer from Boerhaave syndrome, an oesophageal rupture caused by forceful vomiting in an unrelaxed oesophagus. Fluoroscopy demonstrates contrast medium leakage, most often in the lower third in the left lateral position, submucosal contrast retention, or oesophagopleural fistula. In more than 10% of patients, contrast swallow can lead to a false negative result,^87^ explaining the increasing use of CT oesophagography.

## Role of radiology in the interdisciplinary management

Depending on the clinical symptoms, patients may undergo different tests to make the correct diagnosis without following a precise algorithm. Within the widening field of diagnostic modalities, the radiologist’s interpretation of oesophageal fluoroscopy is crucial for further therapeutic decision-making. The complex symptomatology in patients with swallowing disorders and the broad spectrum of underlying pathologies requires close collaboration within the multidisciplinary swallowing team. In dedicated interdisciplinary meetings such as a dysphagia board, fluoroscopic findings can be discussed, and further diagnostic and treatment possibilities can be agreed on to achieve the best patient outcomes.

## Recent developments and future perspectives

Novel techniques have been proposed to improve the interpretation of fluoroscopic swallowing studies and increase quantitative assessment of pathophysiological findings. These include, for example convolutional neural networks for segmentation of image sequences to quantify oesophageal function and estimate oesophageal wall stiffness^88^ or mathematical models to measure temporal changes in geometry.^89^ Combination of fluoroscopy, manometry, and impedance measurement allows information about pressure-flow related to patterns of bolus transit.^90^

## Conclusion

Oesophageal fluoroscopy provides the advantage of simultaneous assessment of morphology and function, a feature not found in other single clinical and diagnostic tests. It enables a comprehensive evaluation of bolus flow in real-time using custom-tailored techniques. Performing fluoroscopy requires a skilled operator with clinical experience. To gain this experience, it is necessary for dedicated fluoroscopists to train fellows in abdominal imaging to improve their interpretive skills and to perform a sufficient number of examinations to acquire these relevant skills. Radiologists should consider this technique, as highlighted in this article, as a valuable imaging tool in various clinical indications, to overcome the shortage of experienced gastrointestinal teachers and to change the perception of oesophageal fluoroscopies as technically demanding and labour-intensive.
